# Self-Reported Difficulty with and Assistance Needed by People with Spinal Cord Injury to Prepare Meals at Home

**DOI:** 10.3390/ijerph21111463

**Published:** 2024-11-01

**Authors:** Katherine Froehlich-Grobe

**Affiliations:** Research Department, Craig Hospital, Englewood, CO 80113, USA; kfroehlich-grobe@craighospital.org

**Keywords:** spinal cord injury, obesity, cardiometabolic disease, dietary intake, kitchen

## Abstract

Individuals with spinal cord injury (SCI) experience an increased risk for obesity and cardiometabolic disease. Recommendations to prevent and treat obesity for those with SCI follow those of the US Department of Agriculture to adopt a healthy eating pattern that includes eating a variety of fruits, vegetables, grains, dairy, and protein, plus limiting added sugars, saturated fats, and sodium. Yet, people with SCI eat too many calories, fat, and carbohydrates and too few fruits, vegetables, and whole grains. The study is based on secondary analyses of SCI participants (*n* = 122) who enrolled in a weight loss study to determine how SCI may impact their ability to prepare food at home. We hypothesize those with higher-level spinal injuries (specifically, those with cervical versus those with thoracic or lumbar/sacral injuries) experience significantly greater difficulty and are more likely to rely on others’ assistance to perform meal preparation tasks. Physiologic (weight, BMI, blood pressure, hemoglobin A1c) and self-reported data (demographic plus responses to the Life Habits Short Survey and meal prep items) were collected at baseline and qualitative data were obtained from a subsample after the intervention during phone interviews. Participants’ average age was 50 ± 14.7 years old, they lived with SCI for an average of 13.0 ± 13.1 years, and their average BMI was 32.0 ± 6.5. Participants were predominantly white (76.1%) men (54.1%) who had some college education (76.3%), though only 28.8% worked. A substantial proportion of respondents (30% to 68%) reported difficulty across the 13 tasks related to purchasing and preparing meals, with a proxy reported as the most common assistance type used across all tasks (17% to 42%). Forty-nine percent reported difficulty preparing simple meals, with 29% reporting a proxy does the task. More than half reported difficulty using the oven and stove, though between 60% to 70% reported no difficulty using other kitchen appliances (e.g., coffee machine, food processor, can opener), the refrigerator, or microwave. There was a significant difference in kitchen function by injury level. Those living with cervical-level injuries had significantly greater limitations than those with thoracic-level injuries. Spouses, other family members, and caregivers were most likely to serve as proxies and these individuals exerted both positive and negative influences on respondents’ dietary intake, based on qualitative data obtained during interviews. The results suggest that many people living with SCI experience functional and environmental barriers that impact their ability to prepare food and use kitchen appliances. Future research should examine how SCI-related functional limitations, transportation access, accessibility of the kitchen, ability to use appliances, availability of financial resources, and assistance by others to prepare foods impact people’s ability to follow a healthy eating pattern.

## 1. Introduction

Obesity occurs among between 40–70% [1,2,3,4,5,6] of people living with spinal cord injury (SCI), which poses an increased risk for the cascade of obesity-related chronic health issues [7,8,9,10,11,12,13]. People with SCI experience greater risk than people without SCI [14] for each factor identified in cardiometabolic disease (CMD)—abdominal adiposity, dyslipidemia, hypertension, insulin resistance or glucose intolerance, pro-inflammatory state, and prothrombotic state [15]. Notably, CMD increases the risk for Type 2 diabetes mellitus (T2DM), cardiovascular disease, and mortality [16].

Life expectancy for individuals with SCI is significantly lower than the general population and age-adjusted mortality rates are three times higher among those with SCI than the general population [17,18]. Obesity contributes to two—heart disease and cancer—of the five leading causes of death among those with SCI. SCI mortality rates are 1.9 times higher for heart disease; 1.9 times higher for endocrine, nutrition, and metabolic diseases; 1.7 times higher for stroke; and 1.3 times higher for cancer than the general population [17]. Being obese represents a potentially modifiable factor that can reduce morbidity and mortality rates among those with SCI given the high obesity rates [1,2,19,20]. The body of accumulating evidence has led to increased interest in addressing dietary intake and weight management for those with SCI [14,21,22,23,24,25].

## 2. Address the Significant Needs of Individuals with Disabilities

The Consortium on Spinal Cord Medicine in collaboration with the Paralyzed Veterans of America (PVA) published clinical guidelines in 2018 [14] to identify and manage CMD in those with SCI. The authors state that CMD may be more challenging to treat in people with SCI than in the general population and argue for conducting early assessment and aggressive preemptive care to prevent early morbidity and mortality. The guidelines recommend evaluating all adults for the components of CMD—obesity (based on body composition if possible or a BMI ≥ 22 if relying on this indicator), impaired fasting glucose, prediabetes, diabetes, hypertension, and dyslipidemia before discharge from inpatient rehabilitation or at the earliest opportunity for those already discharged. The guidelines also recommend screening every three years for obesity, prediabetes, diabetes, and dyslipidemia if the initial screen is normal; and annually for hypertension. Notably, lifestyle intervention is recommended as the first line of therapy to manage any components of CMD and the authors offer guidelines for physical activity and nutrition. The nutrition guidelines are based on those of the US Department of Agriculture (USDA), the core elements of which are shown in Table 1.

## 3. Dietary Intake for Those with SCI

Dietary intake studies conducted with SCI samples suggest they have inadequate dietary intake [22,25,26,27,28,29] compared to the USDA’s recommended daily allowance (RDA) in terms of consuming too much fat [26,27,29], carbohydrates [27,30], and calories [22], plus insufficient dairy, fruit [27], vegetables, and whole grains [29]. Nutritional counseling has been suggested for those with SCI [25,27,31,32]. Two nutrition counseling interventions conducted with SCI samples [32,33] have been published, both of which delivered six, dietitian-led educational sessions.

Lieberman and colleagues [33] randomized individuals with SCI to receive (a) the standard of care nutrition intervention, which was a single nutrition lecture or (b) a group-based, six-session educational intervention. The study delivered nutrition education to both acute and chronic SCI participants, with randomization stratified by time since injury and nutrition education for the acute SCI sample occurred during their inpatient rehabilitation stay. Notably, the team has yet to publish results and according to clinicaltrials.gov, the study closed having enrolled just 38% of their targeted sample size. Wood and colleagues reported [34] that 10 of the 15 enrolled participants (67%) completed the 12-week program of six dietitian-delivered one-on-one telehealth sessions. Participants reported making significantly better food choices but did not demonstrate significant changes in nutrition knowledge, weight, or waist circumference.

## 4. Feasibility of Implementing Dietary Guidelines

One overlooked aspect of dietary intake for people with SCI relates to their ability—both financial and functional—to purchase and prepare the types of recommended foods. Several factors may impact individuals’ ability to feasibly purchase and/or prepare healthy foods. People with SCI are less likely to work, with employment rates among working-age adults with SCI ranging from 35% [35] to 52% [36]. Notably, several years elapse between injury onset and return to work for those who do resume employment. It takes an average of 4.8 years for individuals to obtain their first post-injury job and 6.3 years until obtaining their first full-time post-injury job [37]. Thus, many may need to obtain Social Security for income support and Medicaid for health insurance.

Moving onto federal assistance programs typically leads to reduced income and earnings potential and impacts one’s financial resources. Fewer financial resources affect not only one’s food budget but also the resources available for housing and transportation, which can collectively impact access to grocery stores. Additionally, for those who receive personal assistance services (PAS), whether paid via home and community-based waiver programs or privately, paid caregiver time is likely affected by the need to prioritize self-care tasks such as getting in/out of bed, bathing, toileting, and dressing. For individuals with SCI who rely on PAS, their functional limitations may limit their ability to prepare foods (e.g., cutting vegetables, boiling water) or use kitchen appliances (e.g., refrigerator, stove). These issues may impact their ability to follow dietary recommendations, even if they possess the knowledge and desire to consume a healthy diet.

To our knowledge, evidence regarding how SCI influences the functional abilities of those with SCI to follow dietary recommendations is lacking. The purpose of this study is to examine SCI respondent’s self-reported difficulty with and assistance needed for performing tasks related to preparing simple and complex meals, accessing kitchen appliances, and conducting ancillary activities related to meal preparation such as setting the table, serving the food, and washing the dishes. We hypothesize that those with higher-level spinal injuries (specifically, those with cervical versus those with thoracic or lumbar/sacral injuries) will experience significantly greater difficulty and be more likely to rely on others’ assistance to perform these tasks.

## 5. Methods

This study is a secondary analysis of data collected from individuals living with SCI who enrolled in one of two weight loss intervention studies conducted by the author. Screening and enrollment for one study (*n* = 32 SCI participants) occurred over the summer and fall of 2015 and across four separate four-month time periods between 2019–2021 in the second study (*n* = 99 SCI participants). Respondents in both studies completed self-report surveys at baseline during their in-person lab appointment. Surveys were completed using paper and pencil for the first sample, while the second sample completed their surveys on a computer using the REDCap [38] system. Participants provided information regarding their demographic characteristics; completed 13 items from the Life Habits survey about their difficulty with and assistance used to perform food-preparation tasks in the kitchen; and all underwent a physiologic assessment to obtain measures of weight, waist circumference, blood pressure, and hemoglobin A1c. Participants completed 13 questions from the Life Habits survey [39], a tool designed to assess individuals with disabilities quality of participation by asking about the level of difficulty they have and assistance required to perform activities related to meal preparation and clean up, plus the Life Habits 16-item Short Survey to assess participants overall need for assistance in daily life. Qualitative data were obtained anonymously with a random subsample of 42 respondents via phone interviews after completing a weight loss intervention to ask who assists with buying and preparing their food and how those individuals could be included in the intervention program.

The studies were reviewed and approved by the IRB operated by the organization to which the funding was awarded. Participants were recruited for each study in a large southern metropolitan area. Recruitment strategies included sharing flyers with physical medicine and rehabilitation providers at two large rehabilitation hospital settings, regional durable medical equipment suppliers, and the local center for independent living. Those interested in participating contacted the study staff and were screened by phone. Eligibility was based upon participants’ being over 18 years old, living with a mobility impairment for at least 12 months, being overweight or obese as determined by a body mass index of 22 or greater, and receiving medical clearance from their doctor to participate in a lifestyle intervention. The lifestyle intervention targeted weight loss through reducing daily caloric and fat intake and increasing participation in physical activity. Ineligibility was based on not being considered overweight, not having a permanent mobility impairment, having a grade III or IV pressure injury, or having contra-indications their doctor identified for adopting a lifestyle change program.

Data for this study are based on respondent surveys completed at their baseline assessment and after their study completion for qualitative input.

Demographics. The survey asked participants to report on basic demographic data that included age, time with injury, injury level, wheelchair use, sex, race/ethnicity, marital status, education, employment, and annual household income. Participants also reported on the number of people who they live with and receipt of paid and unpaid daily hours of caregiving was reported on by participants in one of the two studies (*n* = 99).

Life Habits survey. The Life Habits survey assesses respondents’ perceived performance in conducting activities of daily living (six domains) and social roles (seven domains) based on rating the level of difficulty they experience and the assistance they use to complete tasks within each domain [39,40,41,42,43]. The developers have created several versions, including a long (242 items) version [39], a short (77 items) version [44], and a 16-item short version [45]. All versions ask respondents to report on the level of difficulty (none, with difficulty, accomplished by a proxy, not accomplished, not applicable), the type of assistance used (none, assistive device, adaptation, human assistance), and their level of satisfaction with their performance. Satisfaction is scored separately from the difficulty and assistance items, which are combined for an accomplishment score. Table 2 indicates how the scoring reflects reported difficulty by type of assistance used, with human assistance deemed the greatest level of assistance needed.

Study respondents completed the 16-item short version plus answered 13 questions from the 77-item short version related to diet (2 items) and meal preparation (11 items). Data from the 16-item short version provide an estimate of participants’ overall level of function, while responses to the diet/meal items provide an estimate of participants’ level of function related to preparing meals. The 13 diet/meal questions asked participants to report on their level of difficulty with and assistance used for (a) planning/preparing food (planning grocery purchases, selecting appropriate foods for meals, preparing simple meals, and preparing complex meals), using five kitchen appliances (stove, oven, microwave, refrigerator, and other kitchen appliances), and ancillary activities to cooking (serving food, setting/clearing the table, and washing dishes by hand and dishwasher).

Physiologic measures. Participants visited the study lab where several physiologic measures were collected before they initiated the weight loss programs. Resting blood pressure was taken after the participants had been sitting quietly for 10 min before any other measures were taken, with an average of two readings recorded. Body weight was measured using a Seca (model #676) wheelchair-accessible scale by having participants weighed in their chairs and then transferred out of their chairs to obtain the wheelchair weight. Participants’ body weight was calculated by subtracting the wheelchair weight from the total weight. Both the person and wheelchair were weighed three separate times to ensure an accurate weight was obtained, and the average of the three weights was used. Waist circumference was measured while participants were lying supine on a mat table once they transferred out of their chairs. A tape measure was used to measure their waist circumference at the level of the umbilicus, with an average of three measures reported. Additionally, a venous blood draw was collected to measure participants’ hemoglobin A1c as an indicator of blood sugar levels over the past three months.

Analyses include conducting basic descriptive statistics, including means for ordinal values and frequencies for values. We also conducted between-group analyses to determine whether there were significant differences between those with different levels of spinal injury of cervical, thoracic, lumbar/sacral, and the unsure groups. Analyses of variance were conducted on continuous and ordinal values, with the Bonferroni adjustment used for post-hoc analyses to determine which groups differed on the variable. Chi-square analyses investigated whether between-group differences occurred across categorical variables and correlations were run to examine whether overall or kitchen functioning as measured on the Life Habits survey was related to weight, BMI, time with injury, age, or hours of daily caregiver assistance (paid and unpaid). Participant responses and quotes from interviews are also presented.

## 6. Results

Demographic data for the 122 participants are shown in Table 3, with data presented for the full sample and across each injury level from cervical (*n* = 54, 44.3%), thoracic (*n* = 53, 43.4%), lumbar/sacral (*n* = 10, 8.2%), and those who were unsure (*n* = 5, 4.1%). The summary data reported here represent those of the overall group. Respondents were middle-aged (50.0 ± 14.7 years old), though there were significant age differences between those with thoracic-level injuries (50.8 ± 13.9) who were significantly older than those with cervical injuries (45.1 ± 13.7), but who were significantly younger than those with lumbar/sacral injuries (64.3 ± 13.8). The sample lived an average of 13.0 ± 13.1 years with SCI, over half (54.1%) were men, and most (84.4%) were wheelchair users. The groups did not differ by time living with SCI or sex, but those with thoracic-level injuries were significantly more likely to use a wheelchair (92.5%) than the cervical-level (81.5%), or lumbar/sacral (50%) groups.

As indicated in Table 3, demographic data for race/ethnicity, marital status, education, employment status, and income did not differ by injury level. Most (76.1%) participants were White, though 16.4% were Black, some (3.3%) reported being multiracial, and 9.0% were of Hispanic ethnicity. Fewer than half (46.5%) were married or living with a significant other, 19.3% were separated or divorced, and 30.7% reported being single. Participants were highly educated, with more than one-third (37.3%) having earned an undergraduate or graduate degree and another third (36.4%) having attended some college or earning an associate’s degree. Over one-quarter (28.8%) were employed full or part time and more than one-third of respondents (38.0%) reported being in the highest income category (≥$75,000) while one-quarter (25.9%) were in the lowest category (<$25,000).

Table 3 also indicates that participants lived with an average of 2.1 ± 1.7 people. Of the 99 people asked about their receipt of caregiving, 86 (86.9%) reported receiving an average of 2.4 ± 4.5 h of paid caregiving daily and 4.8 ± 7.8 h daily of unpaid caregiving. One-quarter (25.6%) of participants reported that if money were not a consideration, they would benefit from receiving another 4.8 ± 4.4 h of caregiving daily. Caregiving hours did not differ by injury group.

The physiologic data obtained at baseline (Table 3) show that participants’ average body mass index (BMI) was 32.0 ± 6.5, weight was 212.2 ± 47.1 pounds with an average waist circumference of 45.9 ± 14.0 inches. Participants’ average blood pressure and A1c values were in the normal ranges (<5.7%). There were no significant differences by injury level for weight, waist circumference, or A1c values, though blood pressure differed significantly by injury level. Systolic blood pressure was significantly lower for the cervical group (104.8 ± 24.3) than both the thoracic (126.8 ± 16.9) and lumbar/sacral (131.0 ± 21.4) groups. Diastolic pressure was significantly lower for the cervical group (67.3 ± 15.8) than for the thoracic group (74.5 ± 10.2). Those with cervical-level injuries (30.2 ± 5.7) had significantly lower BMI than those with thoracic (33.2 ± 6.8) and lumbar/sacral-level injuries (35.4 ± 5.3).

**Figure 1:**
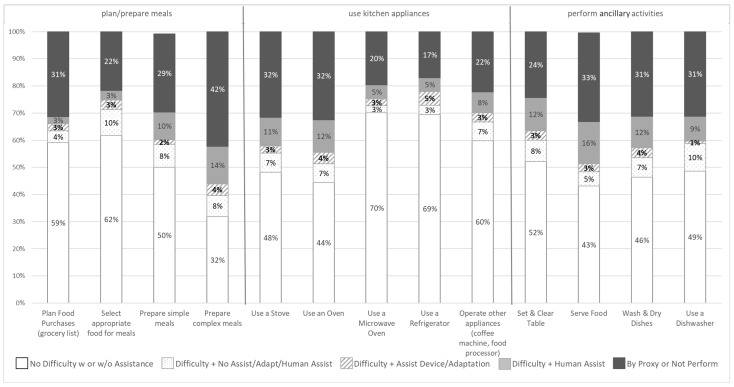
Self-reported level of difficulty and assistance used to perform each task for the full sample (*n* = 122).

Kitchen Access. Data regarding participants’ self-reported difficulty with and assistance used to perform 13 tasks related to preparing meals, accessing kitchen appliances, and conducting ancillary food preparation tasks in the kitchen are presented as percentages in Figure 1 for the full sample. The figure depicts a stacked column for each task that reflects the percentage of participants (out of 100%) who reported no difficulty performing the task (white bar outlined in gray) and various shades and patterns indicate the type of assistance used to perform the task, from using a proxy to using no assistance. The four shades and patterns reflect the assistance used to perform the task: (1) no assistive device or adaptation despite reporting difficulty (white background with light gray dots), (2) an assistive device or adaptation (white background with light gray lined pattern), (3) human assistance only or human assistance plus assistive devices or adaptations (lighter gray), or (4) a proxy or not performed at all (dark gray). Gray lines on the figure visually distinguish tasks that fall into one of three categories related to planning/preparing meals (four items), accessing/using kitchen appliances (five appliances), and performing ancillary activities related to food prep (four tasks).

Nearly half (49%) of the sample reported difficulty preparing simple meals, with 29% reporting that a proxy completes the task and 68% reported difficulty preparing complex meals of whom 42% reported a proxy completes that task. Well over half of the sample reported no difficulty planning (59%) or selecting (62%) their food purchases, though a sizeable percentage relied on a proxy to do those activities (plan 31% and select 22%). More than half of respondents reported difficulty using the stove (52%) and oven (56%) and 32% indicated that a proxy does those tasks. Most report no difficulty using the refrigerator (69%) or microwave (70%), though a proxy is the most common assistance used (17% and 20%, respectively) by those reporting difficulty. Half or more respondents report difficulty performing ancillary activities to preparing meals such as serving food (57%), washing/drying the dishes (54%), or using the dishwasher (51%) and between one-quarter to two-thirds indicate a proxy performs these tasks.

Comparing participant responses by injury level (cervical, thoracic, lumbar/sacral, and unsure) indicates that those with higher-level injuries report more difficulty and rely on greater assistance on the Life Habits survey of access in the kitchen (Table 4). Those with cervical-level injuries reported greater limitations than those with thoracic-level injuries for all activities except planning and selecting food to purchase for meal prep. Those with lumbar/sacral injuries reported significantly greater limitations than those with thoracic-level injuries on four activities related to setting the table, serving food, washing/drying dishes, and preparing complex meals. There were no significant differences between those with thoracic and lumbar/sacral injuries across the nine other meal prep tasks. Notably, there were no significant differences in task accomplishment based on sex.

Correlational analyses (Table 5) indicate that lower functioning in the kitchen was significantly related to greater hours of unpaid (*r* = −0.417) and paid (*r* = −0.293) daily assistance and lower overall Life Habit function (*r* = 0.268). Higher function in the kitchen demonstrated a significant, though small relationship to younger age (*r* = 0.236) and fewer years with injury (*r* = 0.223). Neither weight nor BMI were related to kitchen functioning, though both showed a positive and small significant relationship to overall Life Habit functioning (*r* = 0.264 and *r* = 0.296, respectively). Additional analyses indicate that functioning in the kitchen is significantly related to employment and income. Employed respondents reported significantly better kitchen function (7.0 ± 2.4) than those not working (5.1 ± 2.9) and those in households with annual incomes between $25,000 and $74,999 reported higher functioning in the kitchen (6.9 ± 2.5) than those in households with <$25,000 (5.3 ± 3.0) or ≥$75,000 (5.4 ± 2.9).

Interview Data. Interviews with a subsample of 42 participants indicate that most participants (67%) reported doing the grocery shopping themselves, with spouses (31%), family members (21%), and caregivers (14%) also buying the groceries. Almost everyone (84%) who relies on someone else to purchase groceries reported having input on what is bought. Most (67%) also reported doing the cooking, though spouses (40%), other family members (21%), caregivers (17%), and others (12%) were reported to cook for those who do not. While one individual reported having *“total control; telling the caregiver exactly how they want things cooked and seasoned”* another stated, *“if the caregiver’s mind was set on making something, they would make it and I had no input.”* When asked if others in the home (whether family member or caregiver) influenced their dietary intake, positive comments included, “*together we make a consistent effort to eat healthy—more salads, more cooking at home and less eating out, and we avoid bringing junk food home*”; *“[it is] helpful to have people that are health conscious and keep you accountable*”; and *“My daughter is a role model for me and I am able to learn from her healthy lifestyle….[she] is aware of what she eats….plus aware of how to prepare foods and use portion control”.* Whereas negative influences included, “*caregiver brought home temptation foods and sometimes I broke down and ate them; they are not always the best influence in maintaining a healthy lifestyle,”* and *“My sister does not eat healthy, which makes it difficult and sometimes she influences me to eat things that I shouldn’t”.*

When asked about kitchen modifications they made or what adaptive equipment they used in the kitchen, several participants indicated that their spouse/mother/caregiver does all the cooking so this was not necessary. One person noted, *“I do not want to disrupt the kitchen environment that my mother is used to”.* Though one person noted they *“moved to a completely accessible apartment that has lower counters, a side-by-side fridge and freezer, and stove with controls in front”*. Types of accommodations included *“using a net for the big freezer to be able to reach everything”* and *“moved locations of plates and other kitchen items to the front of cabinets to be more accessible”*. Assistive devices individuals reported using included gripper pads, bowls and plates with lips, adaptive silverware with larger grips, modified knives with large handles, a rocking knife, a specialized cutting board with a knife attached, modified scissors, an electric can opener, and a reacher to get items from the refrigerator or out of cabinets.

Participants were also asked to reflect on how best to include others who are involved in selecting and preparing the foods they eat within the dietary intervention. The vast majority (81%) of interviewees stated that it would be helpful to have several sessions specifically designed for caregivers to attend in which they could be involved in preparing easy, but healthy meals and learn about how food influences health. Suggestions also included creating and sharing grocery shopping lists that caregivers could use, providing recipes for easy and healthy meals, as well as providing caregivers education about how to read food labels, understand low-calorie and low-fat gram options, and practice how to improve communication around food choices and preparation techniques.

## 7. Discussion

These results offer initial evidence that many people who live with SCI experience environmental and functional barriers that limit their ability to prepare food and proxies are the most common method used to perform these tasks. Half to two-thirds of the sample, respectively, reported difficulty preparing simple or complex meals, with most using proxies (29% and 42%, respectively) or human assistance (10% and 14%, respectively) to prepare these meals. Most respondents (~70%) reported no difficulty using the refrigerator and microwave although more than half reported difficulty using the oven and stove (56% and 52%, respectively). Additionally, over half of respondents reported difficulty conducting ancillary, though critical components of meal prep—serving food, cleaning the dishes, and using the dishwasher, with one-third relying on proxies for those tasks. The findings also revealed that people with cervical-level injuries experienced significantly greater limitations in preparing meals and accessing kitchen appliances than those with thoracic-level injuries, but limitations did not differ based on sex. Yet, greater weight was not related to lower kitchen functioning in this sample. Notably, the average BMI of the sample placed participants well within the obesity category (32.0 ± 6.5) and may hint toward a ceiling effect.

Self-reports of the difficulty experienced and assistance used by this SCI sample offer preliminary insights indicating that many individuals with SCI rely on other people to perform or assist performing various functions to select, prepare, cook, and serve their food. Relying on others may have implications for people’s ability to follow dietary recommendations, based on an array of factors that include people’s financial resources, nutritional knowledge, cooking abilities, and time available of those who provide assistance. Qualitative information gathered on a small subsample during phone interviews demonstrates that those who assist with shopping for groceries and preparing food typically live with or regularly assist the person with SCI and include spouses, other family members, caregivers, and others. Additionally, those interviewed reported that these individuals have both positive and negative influences on their dietary intake. Recommendations offered to improve the influence these individuals have on their dietary intake included providing nutrition knowledge such as how to read food labels, identifying low-fat options, and using techniques to prepare food in more healthy ways (grill, bake, swap ingredients for lower-fat/calorie options). Most of those interviewed also suggested bringing individuals who do the cooking onsite to participate in hands-on demonstrations that teach new techniques and allow them to make an easy and healthy meal together.

## 8. Limitations and Conclusions

Several study shortcomings impact our knowledge about the limitations individuals with SCI face in preparing meals. Data were not collected regarding whether or how the issue of food costs impacted buying choices. This has become an important issue for all Americans due to inflation in the post-COVID economy, which has dramatically increased food prices. Fewer than one-third of the study respondents were employed and household income is an indicator of financial resource availability. In this highly educated sample, only 38% reported a household income above the US median income of $74,580 [46] while 25% reported a household income of less than $25,000. Thus, we were unable to determine if and how income may have impacted their ability to purchase fresh fruits and vegetables or the type and amount of protein they buy.

The study did not assess respondents’ social support, social networks, or mental health, which may all contribute to their dietary intake and influence over their dietary choices and decisions. People are influenced by the dietary intake of those with whom they spend time [47] and emerging evidence suggests that dietary intake also affects the gut microbiome and mood states [48]. Another limitation is the lack of a control or comparison group of individuals who have not enrolled in a weight loss trial and are not motivated to make dietary changes.

Other limitations that impact the generalizability of results to the broader population of Americans living with SCI should be noted. First, this was not a random sample of individuals with SCI but rather were individuals who had enrolled in a weight-loss study and were motivated to initiate dietary change. Second, the respondents all resided in a large metropolitan area of the southern U.S. and their responses may not reflect those who live in more rural areas where there may be more limited access to grocery stores and transportation. Third, while the sample included a sizable proportion of Black Americans (16%), respondents were predominantly non-Hispanic white and more were college-educated (46%) than the American population. Thus, the sample is less diverse than exists nationally and likely has greater knowledge about nutrition.

The limitations observed in this study point to the relevance of investigating several issues in future studies. Further study is warranted regarding the impacts that financial resources or lack of financial resources have upon the types of food purchased and specifically the choices individuals make regarding whether they buy fruits and vegetables and whether these are purchased fresh, frozen, or canned. An additional potentially useful question to examine is how financial resource availability impacts people’s decision-making to purchase premade or packaged foods (frozen lasagna, macaroni and cheese, chicken strips, deli meats, etc.) or more basic ingredients to make meals (lean beef, lean chicken, rice, etc.). Those with limited incomes and limited support for personal assistance may lean toward buying premade meals as they require less time and effort to prepare and can be lower cost, but these options typically contain more sodium and fat.

Greater information should also be gathered on how individual factors such as educational level, region of the country where they reside, and/or racial and ethnic status play a role in the foods and cooking techniques that people prefer and are more likely to eat. Regional cuisines may incline people to prefer foods that are more or less healthy such as freshwater fish (bass, trout, catfish), seafood (salmon, shrimp, crab, lobster), wild rice, asparagus, okra, greens, barbecue, fried foods, heavy sauces, cheesy main dishes (alfredo, macaroni and cheese, enchiladas) and thus impact what people commonly eat. Racial/ethnic status may also impact the types of protein sources, vegetables used, and cooking styles (frying, grilling) that people are most used to eating. It will be useful to know how these factors impact people’s dietary intake and how those factors interact with individuals’ level of function and ability to access kitchen appliances to prepare food.

In conclusion, this study offers preliminary evidence that people with SCI experience numerous factors that may impact their dietary intake such as their injury level, accessibility within the kitchen, access to assistive devices and caregivers, and influences of others’ dietary intake in the home. We believe, and the participants suggested, that these factors should be addressed when developing and implementing feasible dietary interventions for people with SCI to follow. To our knowledge, this is the first study to investigate the realities that individuals with SCI experience in being able to shop, prepare, cook, and clean after cooking due to difficulties they face performing the array of tasks required to eat healthy foods on a daily basis. Improving the cardiometabolic health of individuals living with SCI will require understanding how the lived experiences of people with SCI impact their daily choices in balancing their need for assistance with activities of daily living alongside other critical instrumental activities of daily living and how financial constraints may further influence these decisions. Future studies should investigate whether and how specific factors impact the dietary intake of people with SCI including the resources relied upon for obtaining and preparing their daily food (e.g., food benefits like the supplemental nutrition assistance program, Meals on Wheels, food banks); the array of people who assist with preparing meals; people’s access to transportation, grocery stores, and healthy food options (e.g., fresh produce and healthy proteins); the relationship of social support and mental health to dietary intake; and the knowledge and advocacy skills helpful in preparing or directing others to prepare healthy meals. Future studies should also investigate these issues in populations who have less education, who reside within rural and smaller suburban environments where access to transportation and grocery stores is more limited, and who are more racially/ethnically diverse.

## Figures and Tables

**Table 1:** USDA Nutrition Guidelines for 2020–2025.

2020–2025 USDA Guidelines for Adults
1. Follow healthy eating patterns for each life stage
2. Customize and enjoy food and beverage choices to reflect personal preferences, cultural traditions, and budgetary considerations
3. Meet food group needs with nutrient-dense foods and beverages and stay within calorie limits
Vegetables of all types (dark green; red and orange; beans, peas, and lentil; starchy; and other vegetables)
Fruits, especially whole fruit
Grains, at least ½ of which are whole grains
Dairy (fat-free or low-fat milk, yogurt, cheese)
Protein (lean meats, poultry; eggs; seafood; beans, peas, and lentils; and nuts, seeds, soy)
4. Limit foods and beverages higher in added sugars, saturated fat, and sodium, and limit alcoholic beverages
Nutrient-dense foods should comprise 85% of daily calories
Limit added sugars to <10% daily calories from sugar-sweetened drinks, breakfast cereal/bars, candy, desserts,
Limit saturated fat to <10% daily calories from high-fat meat; full-fat dairy products; butter; oils from coconut, palm kernel, and palm and replace with unsaturated fats, particularly polyunsaturated and monosaturated fats (canola, corn, olive, peanut, safflower, soybean, and sunflower)
Limit sodium to ≤2300 mg daily by eating less prepackaged foods (canned soup, chips, deli lunch meat) and dishes like burgers, tacos, rice, pasta, and pizza, in favor of cooking at home more, using lower/reduced sodium products and use spices rather than salt to add flavor

**Table 2:** Scale of accomplishment for performing Life Habits items.

Score	Level of Difficulty	Type of Assistance
9	Performed with no difficulty	No assistance
8	Performed with no difficulty	Assistive device (or adaptation)
7	Performed with difficulty	No assistance
6	Performed with difficulty	Assistive device (or adaptation)
5	Performed with no difficulty	Human assistance
4	Performed with no difficulty	Assistive device (or adaptation) & human assistance
3	Performed with difficulty	Human assistance
2	Performed with difficulty	Assistive device (or adaptation) & human assistance
1	Performed by a substitute	
0	Not performed	
N/A	Not applicable	

Note: Lower scores indicate lower levels of function.

**Table 3:** Demographic and physiologic data of SCI participants and by injury level.

Demographics	All		Cervical Level		Thoracic Level		Lumbar/Sacral		Unknown		*p* Value	Effect Size
	*n* = 122		*n* = 54		*n* = 53		*n* = 10		*n* = 5			
Age, mean ± SD	50.0	±14.7	45.1 ^b^	±13.7	50.8 ^a,c^	±13.9	64.3 ^b^	±13.8	61.4	±12.1	0.000	0.148
Time with injury, mean ± SD	13.0	±13.1	12.5	±12.4	14.0	±14.2	12.8	±14.1	7.4	±6.4	0.743	0.011
Male sex	66	(54.1%)	36	(66.7%)	25	(47.2%)	3	(30.0%)	2	(40.0%)	0.066	na
Wheelchair user	103	(84.4%)	44	(81.5%)	49	(92.5%)	5	(50.0%)	5	(100%)	0.005	na
No. people living in the home	2.1	±1.7	2.2	±1.9	1.9	±1.4	1.7	±1.3	3.3	±2.1	0.368	0.028
Paid caregiving hrs/day ^1,2^	2.4	±4.5	3.6	±4.5	1.2	±3.6	2.2	±6.7	1.3	±2.3	0.149	0.063
Unpaid caregiving hrs/day ^1,2^	4.8	±7.8	5.5	±7.2	3.5	±7.5	8.7	±10.0	7.0	±11.3	0.325	0.028
Use more caregiving hrs/day ^3^	4.8	±4.4	6.7	±5.6	3.0	±1.5	6.0	±0.0	2.0	±0.0	0.254	0.208
Physiologic Variables												
Body weight (lbs)	212.2	±47.1	209.4	±42.7	215.7	±53.5	213.6	±33.8	202.3	±54.5	0.873	0.006
Body mass index	32.0	±6.5	30.2	±5.7	33.2	±6.8	35.4	±5.3	31.9	±10.0	0.037	0.070
Systolic BP (mm Hg)	117.1	±23.2	104.8 ^b,c^	±24.3	126.8 ^a^	±16.9	131.0 ^a^	±21.4	112.7	±9.8	0.000	0.231
Diastolic BP (mm Hg)	70.8	±13.5	67.3 ^b^	±15.8	74.5 ^a^	±10.2	71.9	±15.1	65.8	±3.6	0.042	0.069
Waist circumference (in)	45.9	±14.0	46.3	±19.7	45.5	±7.3	47.3	±5.9	44.6	±7.4	0.974	0.002
Hemoglobin A1c	5.4	±1.0	5.3	±1.0	5.4	±0.6	5.9	±1.9	6.4	±1.1	0.070	0.060
Race/Ethnicity												
White/Caucasian	93	(76.1%)	42	(77.8%)	43	(81.1%)	6	(60.0%)	2	(50.0%)	0.119	
Black/African American	20	(16.4%)	10	(18.5%)	5	(9.4%)	3	(30.0%)	2	(50.0%)		
Asian	1	(0.8%)	1	(1.9%)	0	(0.0%)	0	(0.0%)	0	(0.0%)		
American Indian/Alaskan Native	2	(1.6%)	0	(0.0%)	1	(1.9%)	1	(10.0%)	0	(0.0%)		
More than one race	4	(3.3%)	0	(0.0%)	4	(7.5%)	0	(0.0%)	0	(0.0%)		
Unknown/Missing	2	(1.6%)	1	(1.9%)	0	(0.0%)	0	(0.0%)	0	(0.0%)		
Hispanic Ethnicity	11	(9.0%)	2	(3.7%)	7	(13.2%)	1	(10.0%)	1	(9.1%)	0.638	
Marital status												
Single	35	(30.7%)	18	(36.0%)	15	(29.0%)	1	(11.1%)	1	(25.0%)	0.081	
Married/Living w sign. other	53	(46.5%)	24	(48.0%)	22	(43.1%)	7	(77.8%)	2	(50.0%)		
Separated/Divorced	22	(19.3%)	10	(20.0%)	10	(19.6%)	1	(11.1%)	1	(25.0%)		
Widowed	4	(3.5%)	0	(0%)	4	(7.8%)	0	(0.0%)	0	(0.0%)		
Highest Education												
Some HS/HS Grad	18	(15.8%)	9	(18.0%)	6	(11.8%)	0	(0.0%)	3	(75.0%)	0.052	
Trade/Tech/Vocational	9	(7.9%)	3	(6.0%)	5	(9.8%)	1	(11.1%)	0	(0.0%)		
Some College/Assoc’s Degree	35	(30.7%)	13	(26.%)	20	(39.2%)	2	(22.2%)	0	(0.0%)		
≥Bachelor’s Degree	52	(45.6%)	25	(50.0%)	20	(39.2%)	6	(66.7%)	1	(25.0%)		
Employment status												
Employed (full/part time)	33	(28.8%)	14	(28.0%)	17	(33.3%)	1	(11.1%)	1	(25.0%)	0.252	
Unemployed	46	(39.8%)	24	(48.0%)	18	(35.3%)	2	(22.2%)	2	(50.0%)		
Student/Homemaker/Retired	35	(31.4%)	12	(24.0%)	16	(31.4%)	6	(66.7%)	1	(25.0%)		
Household income ^4^												
Less than $25,000	28	(25.9%)	13	(27.7%)	12	(25.0%)	1	(11.1%)	2	(50.5%)	0.396	
$25,000 to $49,999	22	(20.4%)	7	(14.9%)	12	(25.0%)	2	(22.2%)	1	(25.0%)		
$50,000 to $74,999	17	(15.7%)	5	(10.6%)	11	(22.9%)	1	(11.1%)	0	(0.0%)		
≥$75,000	41	(38.0%)	22	(46.8%)	13	(27.1%)	5	(55.6%)	1	(25.0%)		
^1^ Caregiving questions asked of 99 respondents, 86 (86.9%) reported using paid caregivers												
^2^ Sample for caregiving hours questions		*n*	38		36		9		3			
^3^ Sample about benefit of more hours (*n* = 22)		*n*	10		9		1		2			
^4^ Sample size = 78												

^a^ Cervical sample, ^b^ Thoracic sample, ^c^ Lumbar sample.

**Table 4:** Responses to the Life Habits survey items from the long-form kitchen questions and the summary of the Life Habits Short Survey.

Variables	Full Sample (*n* = 122)		Cervical Level (*n* = 54)		Thoracic Level (*n* = 53)		Lumbar/Sacral (*n* = 10)		Unsure (*n* = 5)		*p*-Value	Effect Size
	Mean SD		Mean SD		Mean SD		Mean SD		Mean SD			
Life Habits Kitchen Survey	5.9	2.8	5.0 ^b^	2.8	7.0 ^a^	2.4	4.5	3.3	6.0	3.1	0.000	0.134
Plan food purchases (grocery list)	5.8	3.6	5.7	3.7	6.5	3.5	4.2	3.5	3.8	3.3	0.148	0.047
Select food for meals	6.4	3.3	6.0	3.6	7.1	3.0	5.1	3.5	6.0	3.7	0.267	0.035
Prepare simple meals	5.5	3.5	4.3 ^b^	3.4	6.8 ^a^	3.2	4.5	3.7	6.8	3.5	0.002	0.125
Prepare complex meals	4.3	3.5	2.8 ^b^	3.0	6.1 ^a,c^	3.3	2.6 ^b^	3.0	5.0	3.1	0.000	0.215
Use a stove	5.5	3.6	4.0 ^b^	3.5	7.1 ^a^	3.0	4.4	3.8	5.8	3.6	0.000	0.174
Use an oven	5.3	3.6	3.8 ^b^	3.5	6.7 ^a^	3.1	4.4	3.8	5.8	3.6	0.000	0.150
Use a microwave oven	6.9	3.3	5.7 ^b^	3.8	8.4 ^a^	1.5	6.0	3.7	6.2	3.9	0.000	0.161
Use a refrigerator (including a freezer)	7.0	3.1	5.8 ^b^	3.6	8.3 ^a^	1.7	6.0	3.7	7.4	3.6	0.001	0.141
Use other appliances (coffee machine, food processor)	6.4	3.4	5.1 ^b^	3.6	7.7 ^a^	2.4	5.6	4.0	7.4	3.6	0.001	0.144
Set and clear the table	5.9	3.5	4.7 ^b^	3.6	7.4 ^a,c^	2.7	3.5 ^b^	3.3	7.0	3.5	0.000	0.174
Serve food	5.2	3.6	3.8 ^b^	3.5	6.7 ^a,c^	3.1	3.5 ^b^	3.3	5.8	4.4	0.000	0.165
Wash and dry dishes	5.4	3.6	4.1 ^b^	3.5	7.0 ^a,c^	3.1	3.6 ^b^	3.4	6.5	3.8	0.000	0.166
Use a dishwasher	5.7	3.6	4.6 ^b^	3.7	7.2 ^a^	2.8	4.8	4.0	6.5	3.8	0.004	0.120

^a^ Cervical sample, ^b^ Thoracic sample, ^c^ Lumbar sample.

**Table 5:** Correlation analyses between Life Habits and other factors.

Variables	Life Habits Kitchen	Life Habits Short	Hours Unpaid Assist	Hours Paid Assist	Age (Years)	Years with Injury	Weight (lbs)	BMI
Life Habits Kitchen (*n* = 122)	1							
Life Habits Short (*n* = 108)	0.268 **	1						
Hours unpaid assist (*n* = 86)	−0.417 **	−0.110	1					
Hours paid assist (*n* = 86)	−0.293 **	−0.078	0.249 *	1				
Age (years) (*n* = 122)	0.236 **	−0.054	−0.124	−0.278 **	1			
Years with injury (*n* = 121)	0.223 *	0.135	−0.244 *	−0.175	0.286 **	1		
Weight (lbs) (*n* = 120)	−0.114	0.264 **	−0.071	0.025	−0.035	−0.163	1	
BMI (*n* = 120)	−0.058	0.296 **	0.007	−0.136	0.032	−0.128	0.416 **	1

* Significant at the 0.05 level (2-tailed); ** Significant at the 0.01 level (2-tailed).

## Data Availability

The raw data supporting the conclusions of this article will be made available by the authors upon request.
